# Comparative evolutionary histories of fungal proteases reveal gene gains in the mycoparasitic and nematode-parasitic fungus *Clonostachys rosea*

**DOI:** 10.1186/s12862-018-1291-1

**Published:** 2018-11-16

**Authors:** Mudassir Iqbal, Mukesh Dubey, Mikael Gudmundsson, Maria Viketoft, Dan Funck Jensen, Magnus Karlsson

**Affiliations:** 1 Department of Forest Mycology and Plant Pathology, Swedish University of Agricultural Sciences, Box 7026, SE-75007 Uppsala, Sweden; 2 Department of Molecular Sciences, Swedish University of Agricultural Sciences, Box 7015, SE-75007 Uppsala, Sweden; 3 Department of Ecology, Swedish University of Agricultural Sciences, Box 7044, SE-75007 Uppsala, Sweden

**Keywords:** *Clonostachys rosea*, Comparative genomics, Gene expression, Molecular evolution, Mycoparasitism, Nematode-parasitism, Phylogenetic analysis, Proteases

## Abstract

**Background:**

The ascomycete fungus *Clonostachys rosea* (order Hypocreales) can control several important plant diseases caused by plant pathogenic fungi and nematodes. Subtilisin-like serine proteases are considered to play an important role in pathogenesis in entomopathogenic, mycoparasitic, and nematophagous fungi used for biological control. In this study, we analysed the evolutionary histories of protease gene families, and investigated sequence divergence and regulation of serine protease genes in *C. rosea.*

**Results:**

Proteases of selected hypocrealean fungal species were classified into families based on the MEROPS peptidase database. The highest number of protease genes (590) was found in *Fusarium solani*, followed by *C. rosea* with 576 genes. Analysis of gene family evolution identified non-random changes in gene copy numbers in the five serine protease gene families S1A, S8A, S9X, S12 and S33. Four families, S1A, S8A, S9X, and S33, displayed gene gains in *C. rosea*. A gene-tree / species-tree reconciliation analysis of the S8A family revealed that the gene copy number increase in *C. rosea* was primarily associated with the S08.054 (proteinase K) subgroup. In addition, regulatory and predicted structural differences, including twelve sites evolving under positive selection, among eighteen *C. rosea* S8A serine protease paralog genes were also observed. The *C. rosea* S8A serine protease gene *prs6* was induced during interaction with the plant pathogenic species *F. graminearum*.

**Conclusions:**

Non-random increases in S8A, S9X and S33 serine protease gene numbers in the mycoparasitic species *C. rosea*, *Trichoderma atroviride* and *T. virens* suggests an involvement in fungal-fungal interactions. Regulatory and predicted structural differences between *C. rosea* S8A paralogs indicate that functional diversification is driving the observed increase in gene copy numbers. The induction of *prs6* expression in *C. rosea* during confrontation with *F. graminearum* suggests an involvement of the corresponding protease in fungal-fungal interactions. The results pinpoint the importance of serine proteases for ecological niche adaptation in *C. rosea*, including a potential role in the mycoparasitic attack on fungal prey.

**Electronic supplementary material:**

The online version of this article (10.1186/s12862-018-1291-1) contains supplementary material, which is available to authorized users.

## Background

The fungal order Hypocreales includes an extensive range of ecologically diverse species including saprotrophs, plant pathogens, plant endophytes, mycoparasites, and pathogens of insects and nematodes [1]. Many species from different genera are of high economic importance as plant pathogens (*Claviceps*, *Fusarium*), but also as biological control agents against plant pathogenic fungi (*Clonostachys*, *Trichoderma*), nematodes (*Clonostachys*, *Hirsutella*, *Trichoderma*) and insects (*Beauveria*, *Hirsutella*, *Metarhizium*, *Ophiocordyceps*). Many hypocrealean fungi are multi-trophic in nature [2] and show a substantial amount of flexibility in lifestyle [1]. Phylogenetic studies have proposed that several lifestyle transitions have occurred within the Hypocreales [3], which have had a significant impact on the evolutionary history and the genome content of these fungi [4, 5].

Proteases are considered to play an important role in fungal adaption to new environments and ecological niches, especially in biotic interactions [2]. Fungal serine proteases are suggested to have an important role in both pathogenic [6, 7] and mutualistic associations [8, 9]. On the basis of the catalytic mechanism and amino acid sequence similarity, proteases are grouped into families and clans. According to MEROPS database peptidase classification [10], serine proteases of clan SB (subtilases) are classified into two families, subtilisin-like proteases (S8) and serine-carboxyl proteases (S53) [11]. The S8 subtilisin-like proteases are characterized by the presence of an Asp-His-Ser catalytic triad where the Ser residue is essential for activity [12]. This catalytic triad is very similar to the His-Asp-Ser catalytic triad present in the S1 protease family, which is considered a result of convergent evolution [13]. The S8 family is further grouped into two subfamilies S8A and S8B, distinguished in part by differences in their catalytic sites. Most of the characterized subtilisin-like proteases are grouped in the subtilisin S8A subfamily [11] with proteinase K being a well-known representative, produced by the fungus *Engyodontium album* (previously *Tritirachium album*) [14].

Expanded gene sets of subtilisin-like serine proteases are reported from entomopathogenic fungi such as *Beauveria bassiana* [15], *Cordyceps militaris* [16] and *Metarhizium* spp. [17], and nematode-parasitizing fungi such as *Arthrobotrys oligospora* [18], *Drechslerella stenobrocha* [19], *Hirsutella minnesotensis* [20], *Monacrosporium haptotylum* [21], *Pochonia chlamydosporia* [22], and *Purpureocillium lilacinus* [2]. The situation is more complex in mycoparasitic fungi, as expanded gene sets of serine proteases are reported from *Trichoderma atroviride* and *T. virens* [23], but reduced gene sets are reported from *T. longibrachiatum* and *T. reesei* [24]. Another indication of the importance of proteases for mycoparasitism is the low number of orthologous proteases between *T. atroviride*, *T. reesei* and *T. virens* [23, 25], indicative of a rapid birth-and-death type of evolution driven by functional diversification [26]. Strong selective signatures of functional diversification in the S08.005 subtilisin gene family within the order Hypocreales are also reported [5].

Firm evidence for the involvement of subtilisin-like serine proteases in the infection process of the respective host species is less common. Overexpression of the serine proteases Prb1 in *T.* confer (cf.) *harzianum* [27] and Tvsp1 in *T. virens* [28] improved the ability of the mycoparasites to protect cotton seedlings against the pathogen *Rhizoctonia solani*. Furthermore, the serine proteases SprT from *T. longibrachiatum* [29] and PRA1 from *T*. cf. *harzianum* reduced egg hatching of *Meloidogyne incognita* [30], while ThSS45 from *T.* cf. *harzianum* inhibited growth of *Alternaria alternata* [31]. Serine proteases from several different nematode-parasitizing fungi, including *A. oligospora*, *Dactylella shizishanna*, *H. rhossiliensis*, *Lecanicillium psalliotae* and *P. lilacinus*, have been purified and shown to be involved in the degradation of cuticle and progression of the infection process of nematodes [32–37].

The genome sequence of the mycoparasitic and nematode-parasitic fungus *C. rosea* strain IK726 was recently determined [38], but no genome-wide characterization of proteases in *C. rosea* is currently available. However, several *C. rosea* serine protease genes are upregulated during parasitism of the silver scurf pathogen of potato, *Helminthosporium solani* [39]. Furthermore, the serine protease PrC is shown to be involved in infection of nematodes [40], emphasizing the importance to study proteases in this biological control agent.

In this study, we explore the protease gene family in *C. rosea* and related hypocrealean fungi, and test for regulatory and predicted structural divergence between S8A serine protease paralogs. Due to its reported mycoparasitic and nematode-parasitic lifestyle, we hypothesize that adaptive natural selection has resulted in the expansion of the same protease subfamilies in *C. rosea* as in related mycoparasitic and nematode-parasitic species due to convergent evolution. We further hypothesize that the driver for gene family expansion is functional diversification, exemplified by structural and regulatory differences between paralogs in *C. rosea* and a limited number of shared orthologs with related species. We show that serine protease families S1A, S8A, S9X and S33 evolve non-randomly in *C. rosea*, indicating selection for gene gains. Serine protease families S8A, S9X and S33 also evolve non-randomly in the aggressive mycoparasitic species *T. virens* or *T. atroviride*, suggesting that their role is coupled to the mycoparasitic lifestyle. In addition, predicted structural and regulatory differences between *C. rosea* S8A paralogs suggest that functional diversification is the main driving force for the increased gene copy number.

## Methods

### Biomining of fungal proteases

Ten hypocrealean fungi and *Neurospora crassa* (order Sordariales) were included in studying the evolutionary history of protease gene families. Whole-genome nucleotide and protein sequences of *H minnesotensis* (nematode endoparasitic fungus) [20], *H. thompsonii*, *H. sinensis* and *Metarhizium robertsii* (entomopathogenic fungi) [41–43], *T. atroviride*, *T. virens* and *T. reesei* (mycoparasitic fungi) [23, 44], *Fusarium graminearum* and *F. solani* (plant pathogenic fungi) [45, 46], *N. crassa* (saprophytic fungus) [47] and *C. rosea* [38] were retrieved from the National Center for Biotechnology Information (NCBI), Joint Genome Institute (JGI) and an integrated functional genomics database for fungi (Fungi DB). Proteases were identified and classified into families by BLASTp [48] against the MEROPS peptidase (http://www.ebi.ac.uk/merops) database [10], although protease information for *M. robertsii* was retrieved from Hu et al. [42]. The results were manually edited to create a non-redundant set.

### Protease gene family evolution

Different phylogenetic placements of *C. rosea* (Bionectriaceae family) within the Hypocreales order are reported, either as a sister taxon to the Nectriaceae family (*Fusaria*) [38, 49] or as a basal lineage in Hypocreales [50]. Both topologies were therefore retrieved from the literature [20, 38, 49, 50] and used for the analysis of protease gene family evolution. Branch lengths were calculated based on a four-gene alignment including actin, glyceraldehyde 3-phosphate dehydrogenase, DNA-directed RNA polymerase II subunit B and translation elongation factor 1 alpha. Coding gene sequences were retrieved from the respective genome sequences. Each gene was aligned individually using Clustal W [51] in MEGA ver. 6 [52], concatenated and used to calculate branch lengths in MEGA ver. 6. The obtained species phylogeny was calibrated to the fossil record by setting the split between *H. minnesotensis* and *H. sinensis* to 29 million years, as outlined by Lai et al. [20].

Gene family evolution analysis was carried out on protease families that contained ≥2 genes in at least one species and were present in ≥2 species. The program CAFE (Computational Analysis for Gene Family Evolution) ver. 3 [53] was used to test whether gene family sizes were compatible with a stochastic birth and death model, to estimate gene family size in extinct species and to identify lineages with accelerated rates of gene gain or loss. Mutation rate (λ) was estimated from the data and was 0.017.

### Sequence and phylogenetic analyses

Full-length protein sequences were aligned using Clustal W and phylogenetic trees were constructed using maximum likelihood methods implemented in MEGA ver. 6. The WAG + G (Whelan and Goldman) with G (Gamma distribution) amino acid substitution model [54] was optimal and used with gamma distributed rates among sites and partial deletion of gaps. Statistical support for branches was assessed by 1000-iteration bootstrap resampling. Conserved protein modules and features were identified using the conserved domain database (CDD) [55] at NCBI. Identification of signal peptides for targeting proteins to the secretory pathway was performed using simple modular architecture research tool (SMART) [56] and SignalP ver. 4.1 [57].

### Gene tree / species tree reconciliation analysis

Serine protease phylogenetic trees were compared with a rooted species tree where *C. rosea* was considered as a sister taxon to the Nectriaceae family [38, 49] in order to map each node in the gene tree as either a duplication or a speciation event. The software package NOTUNG ver. 2.9 [58] was used for the reconciliation of gene and species trees. The analyses were performed with default parameters i.e. 1 for losses and 1.5 for duplications and with a 90% bootstrap cutoff in order to collapse poorly supported topologies.

### Analysis of molecular evolution

Regions of low amino acid conservation in protease alignments were identified by reverse conservation analysis (RCA), as described by Lee [59]. Regions that contained gaps in more than 50% of the included species were excluded from the analysis. In short, Rate4Site ver. 2.01 was used to calculate the degree of conservation (S score, high scores correspond to low degree of conservation) for each amino acid position using the empirical Bayesian method [60, 61]. A sliding-window average (*n* = 7) of normalized S scores (mean was 0 and standard deviation was 1) was plotted in Excel (Microsoft) (W mean score) and significant peaks were defined by intensity (I) values of 0.5 [59].

Nucleotide sequences were aligned using Muscle [62] implemented in MEGA ver. 6. The rate of non-synonymous (dN) and synonymous (dS) substitutions at each codon and identification of sites evolving under positive or negative selection, was determined using the random effects maximum likelihood models (REL) [63], implemented in the HyPhy software package [64], accessed through the Datamonkey webserver (https://www.datamonkey.org/) [65]. The optimal nucleotide substitution model was assessed as per recommendation when using REL [63, 66], and included the following modifications to the general reversible nucleotide model [67–69]; A↔T: R_AT_, C↔T: R_CT_, G↔T: R_GT_. A Bayes factor value ≥50 (default) was used as an indication of strong positive selection at a site, while values between 10 and 49 were considered to indicate weak support of positive selection [63].

### Homology modelling of a *C. rosea* S8A serine protease

A homology model of a *C. rosea* S8A serine protease (BN869_T00009071) was constructed by using the I-TASSER (Iterative Threading AssEmbly Refinement) (https://zhanglab.ccmb.med.umich.edu) server [70]. The catalytic domain (amino acid 109 to 385) was modelled with a confidence score (C-score) of 1.74 (− 5 to 2, higher is better) [71], an estimated template modelling score (TM score) of 0.96 ± 0.05 (> 0.5 indicates a molecule of correct topology) [72] and an estimated root mean square deviation (RMSD) of 2.6 ± 1.9 Å (lower is better), while the propeptide domain (amino acid 16 to 107) was modelled with a C-score of − 0.42, an estimated TM score of 0.66 ± 0.13 and an estimated RMSD of 4.6 ± 3.0 Å. I-TASSER is a hierarchical approach to protein structure and function prediction, it utilizes a series of predictive and modelling techniques applied on to a protein sequence query. Ligands present in the structure were imported from homologous protein structures; Ca^2+^ ions imported from the Protein Data Bank ID (PDB:ID): 1IC6 [73] and peptide ligand imported from PDB ID: 2HPZ [74]. Figures and analysis were performed using the PyMOL Molecular Graphics System (ver. 2.0 Schrödinger, LLC.).

### Protease activity estimation using milk powder plate assay

Extracellular protease activity of *C. rosea* was estimated using a milk powder plate assay in four different growth media i.e. potato dextrose agar (PDA, Sigma-Aldrich, Steinheim, Germany), malt extract agar (MEA, Sigma-Aldrich, Steinheim, Germany), Czapek Dox agar (CZ, Sigma-Aldrich, Steinheim, Germany) and water agar. The milk powder Petri dishes (9 cm diameter) contained two layers of agar, where the lower layer consisted of one of the growth media and the upper, thinner layer consisted of milk powder agar as described previously [75]. The milk powder agar was made by preparing fat-free milk powder (Semper) and agar separately in distilled water and autoclaving. After cooling to 50 °C, the solutions were mixed to obtain a final concentration of 10 g/l agar and 60 g/l milk powder. A 5 mm diameter agar plug was cut from the actively growing edge of a *C. rosea* strain IK726 culture and transferred to the milk powder plates and incubated for 7 days at 25 °C under dark conditions. Observation of agar clearing zones, indicative of extracellular protease activity, was done after 3, 5 and 7 days post inoculation. Three biological replicates of each treatment were done.

### Experimental setup for protease gene expression analysis

Gene expression of eighteen S8A serine protease genes (*prs1* to *prs18*) in *C. rosea* was assessed by reverse transcription quantitative polymerase chain reaction (RT-qPCR) in two different experiments;

I. Fungal-fungal interaction: *Clonostachys rosea* strain IK726, *Botrytis cinerea* strain B05.10 and *F. graminearum* strain PH-1 were maintained on PDA plates at 25 °C under dark conditions. *Clonostachys rosea* was confronted in a dual plate assay with *B. cinerea* (Cr-Bc), *F. graminearum* (Cr-Fg), and with itself (Cr-Cr), in 9 cm diameter PDA plates and incubated at 25 °C under dark conditions. *Clonostachys rosea* was inoculated 5 days before *B. cinerea* and *F. graminearum* because of differences in mycelial growth rate. The growing front (7 to 10 mm) of the *C. rosea* mycelium was harvested upon contact with *B. cinerea* or *F. graminearum*. Mycelium harvested at same stage from *C. rosea* confronted with itself served as a control treatment. Each treatment was replicated five times.

II. Growth on protein compounds: E-flasks containing 25 ml liquid PDB were inoculated with 2.5 × 10^5^ *C. rosea* strain IK726 conidia/flask and incubated at 25 °C on a rotating shaker (100 rpm) for 4 days under dark conditions. Fungal biomass were separated from the broth by filtration using sterilised Miracloth (EMD Millipore, Billerica, MA) and the mycelia were washed twice with 20 ml sterile water to completely remove the liquid PDB. The washed mycelia were then transferred into new E-flasks, containing 25 ml of water (control) or a water solution of bovine serum albumin (BSA, Sigma-Aldrich, Steinheim, Germany), collagen (Sigma-Aldrich, Steinheim, Germany) or milk powder (Semper). The 1% solutions of BSA and collagen in sterile water were sterile filtrated through a 0.45 μm cellulose membrane (Sarstedt, Nümbrecht, Germany). The 75 g/l milk powder solution was sterilized by autoclaving. E-flasks were incubated at 25 °C on a rotating shaker (100 rpm) for 6 h under dark conditions to induce protease gene expression. Each treatment was replicated five times.

### Nucleic acid isolation and cDNA synthesis

Genomic DNA was extracted from *C. rosea* as described previously [76]. RNA extraction from *C. rosea* samples was carried out using the Qiagen RNeasy kit according to manufacturer instruction (Qiagen, Hilden, Germany). RNA was treated with RNase-free DNase I (Fermentas, St. Leon-Rot, Germany) and concentration was determined spectrophotometrically using a NanoDrop-1000 (Thermo Scientific, Wilmington, DE). One microgram of total RNA was reverse transcribed in a total volume of 20 μl using the Maxima first strand cDNA synthesis kit (Fermentas, St. Leon-Rot, Germany) followed by 12-fold dilution and was stored at − 20 °C until further use.

### Quantitative PCR

Specific PCR primers targeting *C. rosea* S8A serine protease genes were designed using the PrimerSelect software implemented in DNASTAR Lasergene ver. 10 package (DNASTAR Inc., Madison, WI). Primer amplification efficiencies were determined based on amplification of serial dilutions of *C. rosea* genomic DNA or cDNA. Transcript levels were quantified by an iQ5 qPCR system (Bio-Rad, Hercules, CA) using the SsoFast EvaGreen Supermix (Bio-Rad, Hercules, CA) as described previously [77]. After qPCR reactions, melt curve analyses were performed in order to confirm that the signal was the result of a single product amplification. The relative expression levels for protease genes were calculated in relation to the reference gene tubulin [78] by using the 2^–ΔΔ*C*T^ method [79]. Gene expression analysis was performed in five biological replicates, each based on two technical replicates.

### Statistical analysis

Gene expression data were analysed by performing analysis of variance (ANOVA) using a general linear model approach implemented in Minitab® Statistical Software version 18.1 (Minitab Inc., State Collage, PA). Pairwise comparisons were made using the Fisher’s least significant difference (LSD) method at the 95% significance level.

## Results

### Protease gene numbers

Biomining of fungal genomes and comparisons with the MEROPS peptidase database revealed that *C. rosea* contained a total of 576 genes predicted to encode proteases, which were grouped according to the hierarchical classification system [80] (Additional file 1: Table S1). The number of protease genes in *C. rosea* was higher compared with the other mycoparasitic fungi *T. atroviride*, *T. reesei* and *T. virens* (321–478 genes), the entomopathogenic fungi *H. sinensis*, *H. thompsonii* and *M. robertsii* (224–435 genes), and the nematode endoparasitic fungus *H. minnesotensis* (385 genes). The plant pathogenic *F. graminearum* and *F. solani* contained 435 and 590 protease genes, respectively, more comparable with the number in *C. rosea* (Additional file 1: Table S1). The serine protease gene family was found to be the most numerous in *C. rosea* and the other studied hypocrealean fungi, which encompassed ≥48% of all proteases in *C. rosea*, *F. graminearum*, *F. solani*, *T. atroviride* and *T. virens* (Additional file 1: Table S1). The metalloprotease family was the second most numerous, followed by cysteine proteases. The number of predicted protease inhibitor genes followed the pattern of proteases, with the highest number found in *C. rosea* (13 genes) and *F. graminearum* and *F. solani* (11 genes each), while the other studied fungal species contained between 5 and 10 protease inhibitor genes (Additional file 1: Table S2).

### Analysis of protease gene family evolution

**Fig. 1:**
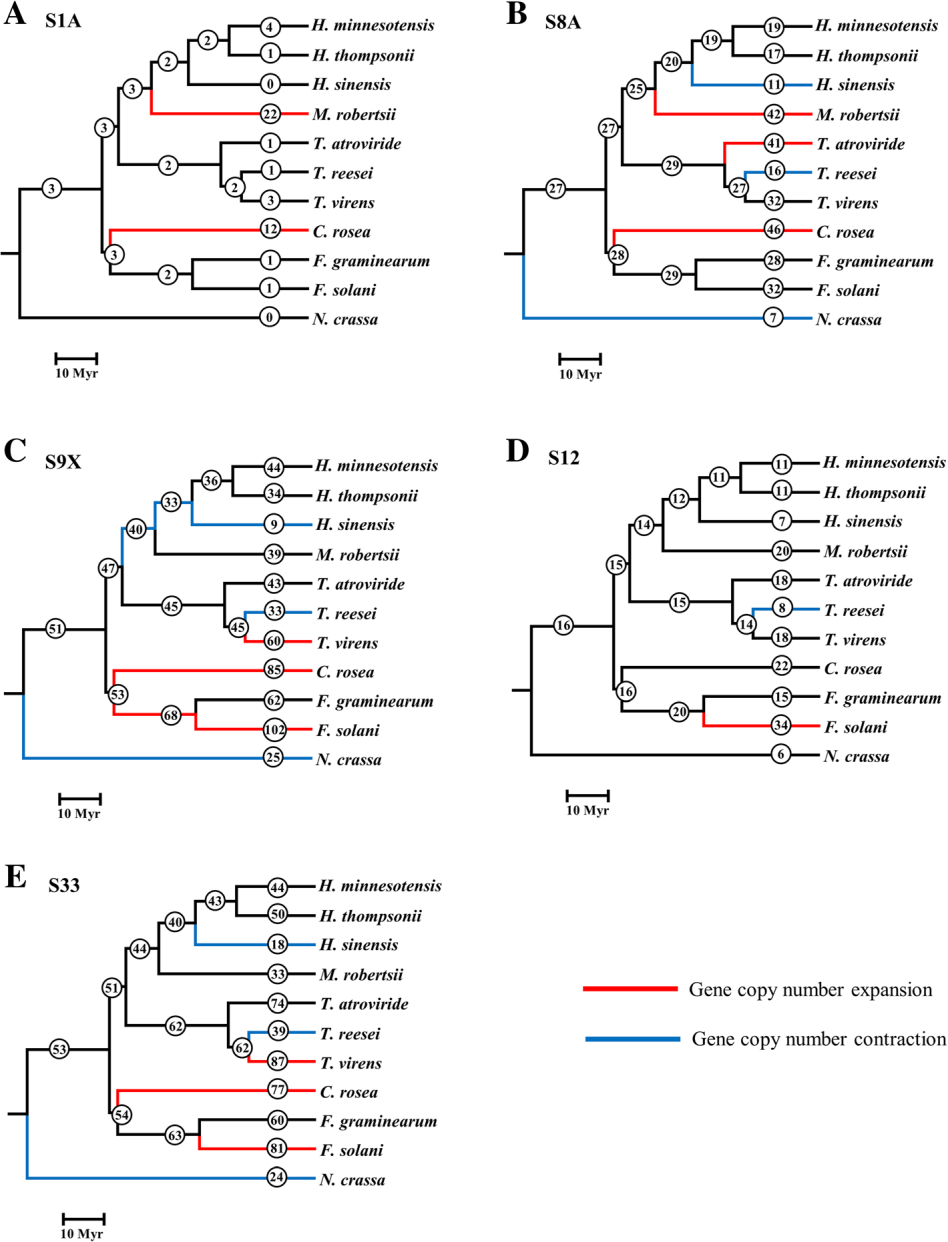
Distribution of serine protease gene gain and loss among fungal species. Circled numbers represent total number of serine protease genes in families (**a**) S1A, (**b**) S8A, (**c**) S9X, (**d**) S12 and (**e**) S33 in extant species and estimates of total number of serine protease genes for ancestral species. Red lineages indicate a significant (*P* ≤ 0.05) expansion of serine proteases genes, whereas blue lineages indicate significant (*P* ≤ 0.05) contractions of serine protease genes

Protease gene family expansions and contractions were analysed using two different phylogenetic trees, differing in the placement of *C. rosea* (Additional file 2: Figure S1). Five serine protease families, S1A, S8A, S9X, S12 and S33, were identified as evolving non-randomly (*P* ≤ 0.05) in both analyses. The serine protease subfamily S1A (type enzyme: chymotrypsin A) was found to be expanded in *C. rosea* and *M. robertsii*, when assuming that *C. rosea* was sister taxon with *Fusaria* (Fig. 1a). The same results were identified when using the alternative phylogenetic placement of *C. rosea,* where *C. rosea* was assumed to be a basal lineage in Hypocreales.

The serine protease subfamily S8A (type enzyme: subtilisin Carlsberg) was expanded in *C. rosea* with 46 genes, compared with an estimated number of 28 genes in the ancestor, *T. atroviride* (from 29 to 41 genes) and in *M. robertsii* (from 25 to 42 genes) (Fig. 1b). Gene losses were detected in *H. sinensis*, *T. reesei* and *N. crassa* (Fig. 1b). The same results were identified when using the alternative phylogenetic placement of *C. rosea*.

The serine protease subfamily S9X was expanded in *C. rosea* (from 53 to 85 genes), *T. virens* (45 to 60 genes), *F. solani* (from 68 to 102 genes), and in the ancestor to *F. graminearum* / *F. solani* (Fig. 1c). Gene losses were detected in *H. sinensis* (from 33 to 9 genes), *T. reesei* (from 45 to 33 genes), *N. crassa* and in the ancestors to *Hirsutella*, and *Hirsutella* / *Metarhizium* (Fig. 1c). In the alternative analysis, similar results were identified except that the gene contraction in the ancestor to *Hirsutella* / *Metarhizium* was not significant (*P* = 0.105).

The serine protease family S12 (type enzyme: D-Ala-D-Ala carboxypeptidase B) was only expanded in *F. solani* (from 20 to 34 genes), but contracted in *T. reesei* (Fig. 1d). The same results were identified when using the alternative phylogenetic placement of *C. rosea*.

The serine protease family S33 (type enzyme: prolyl aminopeptidase) was expanded in gene copy number in *C. rosea* (from 54 to 77 genes), *T. virens* (from 62 to 87 genes) and *F. solani* (from 63 to 81 genes) (Fig. 1e). Non-random gene losses were identified in *H. sinensis* (from 40 to 18 genes), *T. reesei* (from 62 to 39 genes) and *N. crassa* (Fig. 1e). The same results were identified when using the alternative phylogenetic placement of *C. rosea*.

### Phylogenetic and reconciliation analyses of the serine protease subfamily S8A

**Fig. 2:**
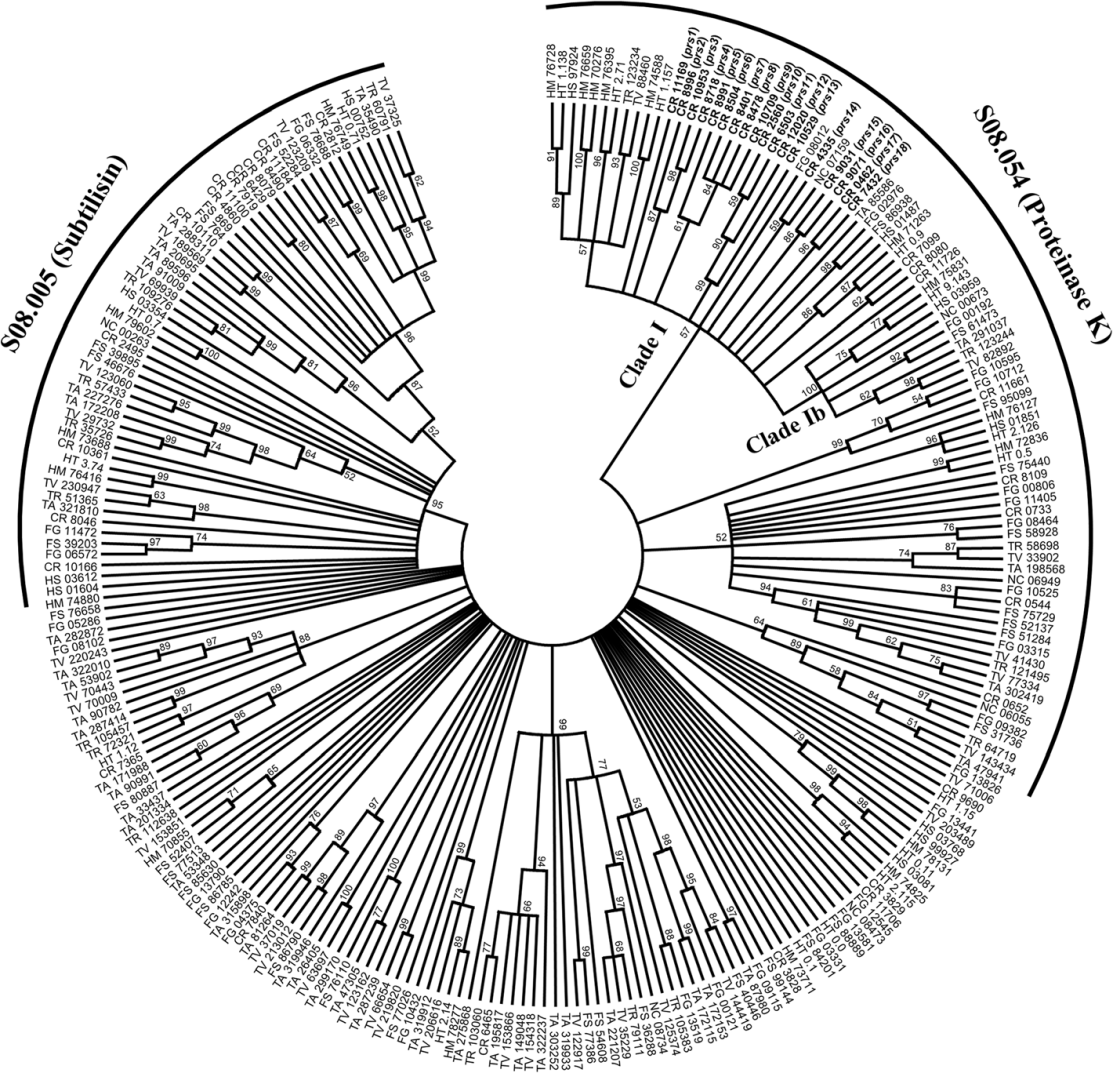
Phylogenetic relationship of S8A serine proteases among fungal species. Amino acid sequences of S8A serine proteases were aligned by Clustal W and used to construct a phylogenetic tree using the maximum likelihood methods in MEGA ver. 6. Branch support values (bootstrap proportions ≥50%) are associated with nodes. Phylogenetic groups referred to as clade I and clade Ib are indicated, as are the S08.005 subtilisin and S08.054 proteinase K groups. Abbreviations: CR = *Clonostachys rosea*, FG = *Fusarium graminearum*, FS = *F. solani*, HM = *Hirsutella minnesotensis*, HT = *H. thompsonii*, HS = *H. sinensis*, TR = *Trichoderma reesei*, TV = *T. virens*, TA = *T. atroviride* and NC = *Neurospora crassa*

**Fig. 3:**
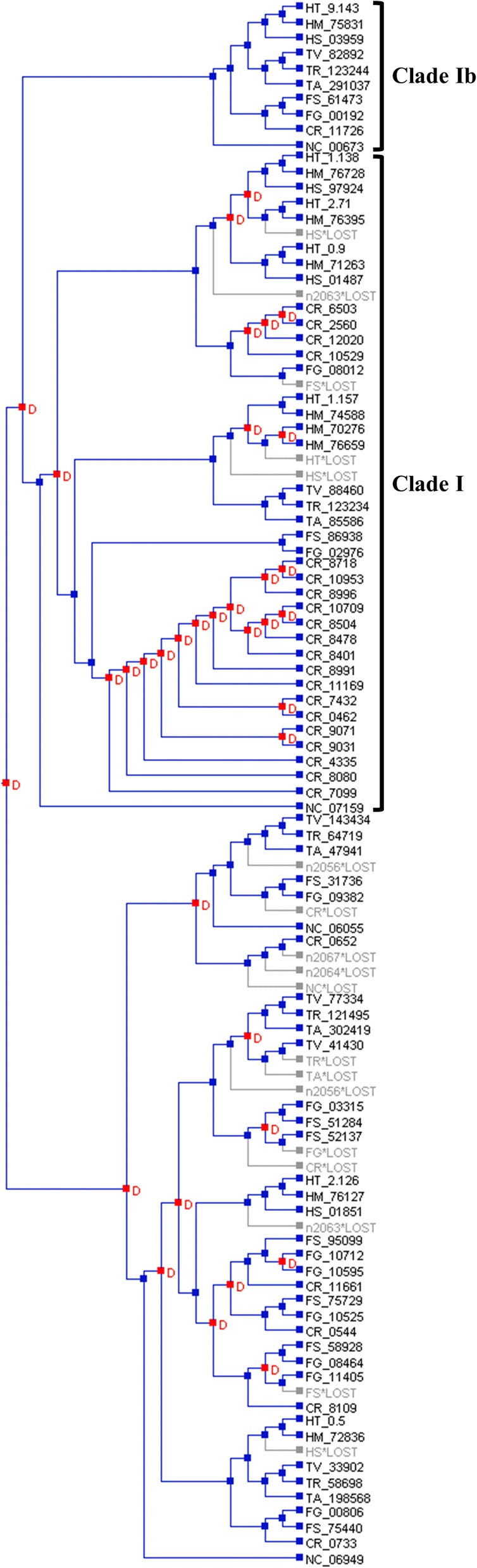
Reconciliation of serine protease S8A subgroup S08.054 (proteinase K) gene tree with the species tree by NOTUNG. Nodes marked in red and the letter D indicates a gene duplication event, while terminal branches marked in grey indicates a gene loss. Abbreviations: CR = *Clonostachys rosea*, FG = *Fusarium graminearum*, FS = *F. solani*, HM = *Hirsutella minnesotensis*, HT = *H. thompsonii*, HS = *H. sinensis*, TR = *Trichoderma reesei*, TV = *T. virens*, TA = *T. atroviride* and NC = *Neurospora crassa*

As S8A serine proteases have been implicated in both mycoparasitism [39] and nematode-parasitism [40] in *C. rosea*, a detailed phylogenetic analysis was conducted on this subfamily. The phylogenetic analysis of 249 S8A serine proteases from nine hypocrealean species and *N. crassa* (Additional file 1: Table S3) revealed low resolution among the deeper branches, although with some supported groups that corresponded to the S08.005 subgroup (subtilisin) and the S08.054 subgroup (proteinase K), respectively [5] (Fig. 2). Reconciling the protease K S08.054 subgroup gene tree with the species tree identified a total of 35 gene duplications and 18 gene losses (Fig. 3). Eighteen gene duplications, but only 2 gene losses, occurred within the *C. rosea* lineage. In contrast, only 3, 4 and 1 gene duplications and 3, 4 and 2 gene losses were identified in the *Fusarium*, *Hirsutella* and *Trichoderma* lineages, respectively. A closer inspection revealed that all 18 *C. rosea* gene duplications took place in a specific phylogenetic group with 57% bootstrap support (here referred to as clade I) within the S08.054 subgroup (Fig. 2), together with representatives from all other included species. With the exception for a small subgroup with 100% bootstrap support that contained one orthologous protein from each of the included species (here referred to as clade Ib), the phylogenetic resolution within clade I was low (Fig. 2). This was exemplified by the fact that only two orthologs present in three different species and no orthologs present in four species or more were found (Fig. 2). The predicted *C. rosea* strain IK726 protein BN869_T00004335 (PRS14) displayed the highest sequence similarity (92% identity) to the previously reported S8A serine protease PrC from *C. rosea* strain 611 [81, 82], suggesting that PRS14 may be orthologous to the PrC protein.

**Fig. 4:**
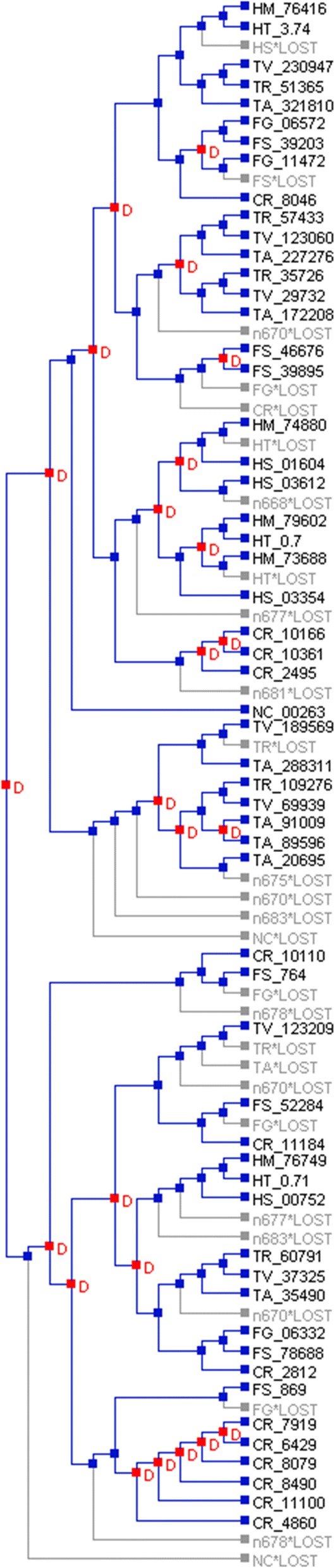
Reconciliation of serine protease S8A subgroup S08.005 (subtilisin) gene tree with the species tree by NOTUNG. Nodes marked in red and the letter D indicates a gene duplication event, while terminal branches marked in grey indicates a gene loss. Abbreviations: CR = *Clonostachys rosea*, FG = *Fusarium graminearum*, FS = *F. solani*, HM = *Hirsutella minnesotensis*, HT = *H. thompsonii*, HS = *H. sinensis*, TR = *Trichoderma reesei*, TV = *T. virens*, TA = *T. atroviride* and NC = *Neurospora crassa*

Reconciliation of the subtilisin subgroup S08.005 gene tree with the species tree identified a total of 24 and 27 gene duplications and losses, respectively (Fig. 4). Again, *C. rosea* stood out with 7 gene duplications, but only 1 gene loss. In contrast, only 2, 3 and 4 gene duplications and 5, 3 and 3 gene losses were identified in the *Fusarium*, *Hirsutella* and *Trichoderma* lineages, respectively. The expansion of S8A genes in *T. atroviride* (Fig. 1) occurred in different phylogenetic groups as compared with *C. rosea* (Fig. 2).

### Sequence divergence and predicted structural changes of *C. rosea* S8A paralogs

**Fig. 5:**
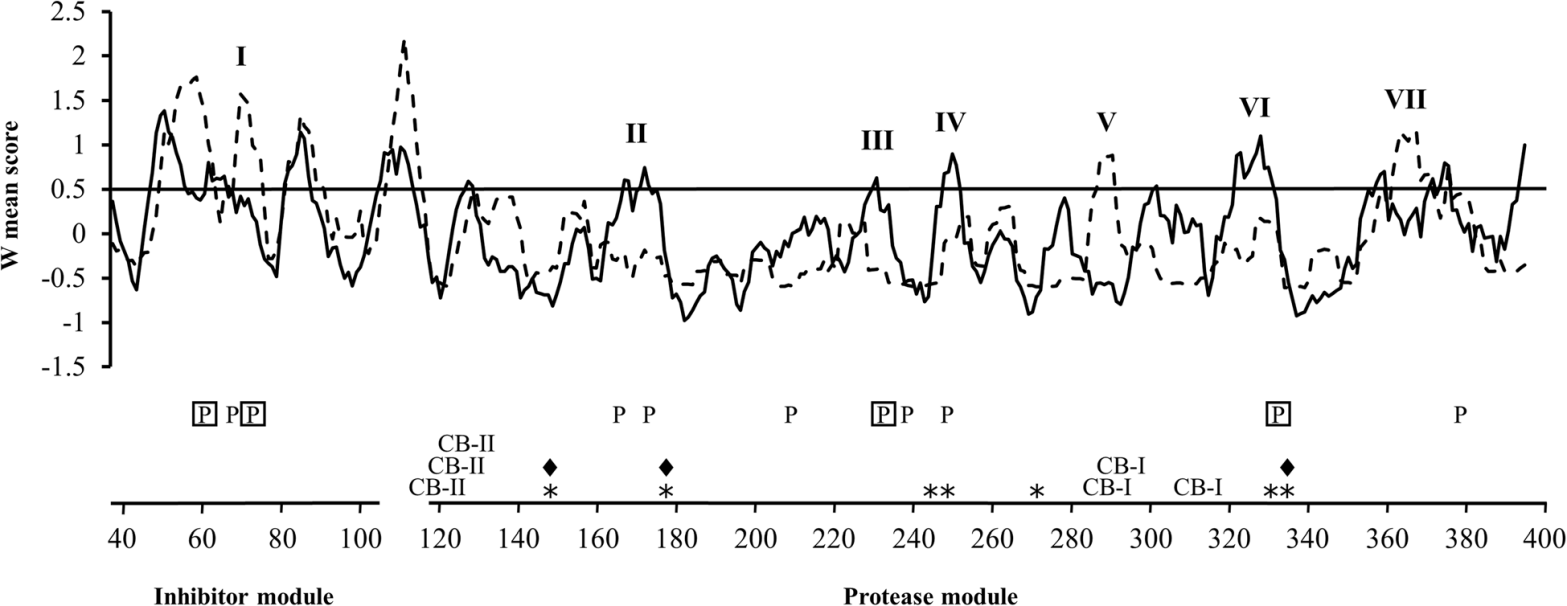
Reverse conservation analysis of two groups of S08.054 serine proteases. Amino acid conservation was estimated using Rate4Site, based on a Clustal W alignment of clade I (solid line) and clade Ib (dashed line) serine proteases (Fig. 2) and plotted as W mean. The y-axis represents arbitrary units while a horizontal line indicates a 0.5 standard deviation cut-off (I). The x-axis represents residue position in reference to *C. rosea* BN869_T00009071 (PRS16), asterisks (*) indicate the positions of predicted active site residues (147, 178, 242, 243, 270, 330, 333), diamonds (♦) indicates predicted catalytic residue positions (147, 178, 333), CB-I and CB-II indicates the positions of the predicted calcium binding-I (284, 286, 309) and calcium binding-II (118, 121, 122) sites, respectively, boxed P indicate residues evolving under strong (Bayes factor ≥ 50) positive selection (62, 72, 231, 328), P indicate residues evolving under weak (Bayes factor 10–49) positive selection (70, 165, 172, 207, 236, 245, 377). Regions that display signs of functional divergence, high variation (*I* ≥ 0.5) in one group in combination with low variation (*I* < 0.5) in the other group, are labelled I through VII

All predicted serine proteases from clade I were predicted to contain an N-terminal signal peptide for secretion pathway targeting (Additional file 1: Table S4). Alignments and RCA analyses were applied to reveal conserved and variable regions between clade Ib and the remaining clade I S8A serine proteases (Fig. 2, Additional file 1: Table S5). Eleven regions with high amino acid diversity (*I* ≥ 0.5) were identified in the clade I S8A serine protease alignments (Fig. 5). Seven of these regions displayed signs of functional divergence, defined as a high variation (I ≥ 0.5) in one group in combination with low variation (*I* < 0.5) in the other group, and were labelled I through VII (Fig. 5). Four of these regions (II, III, IV and VI) displayed sequence diversification between clade I members, including the expanded *C. rosea* paralogs, but were conserved between clade Ib members.

**Fig. 6:**
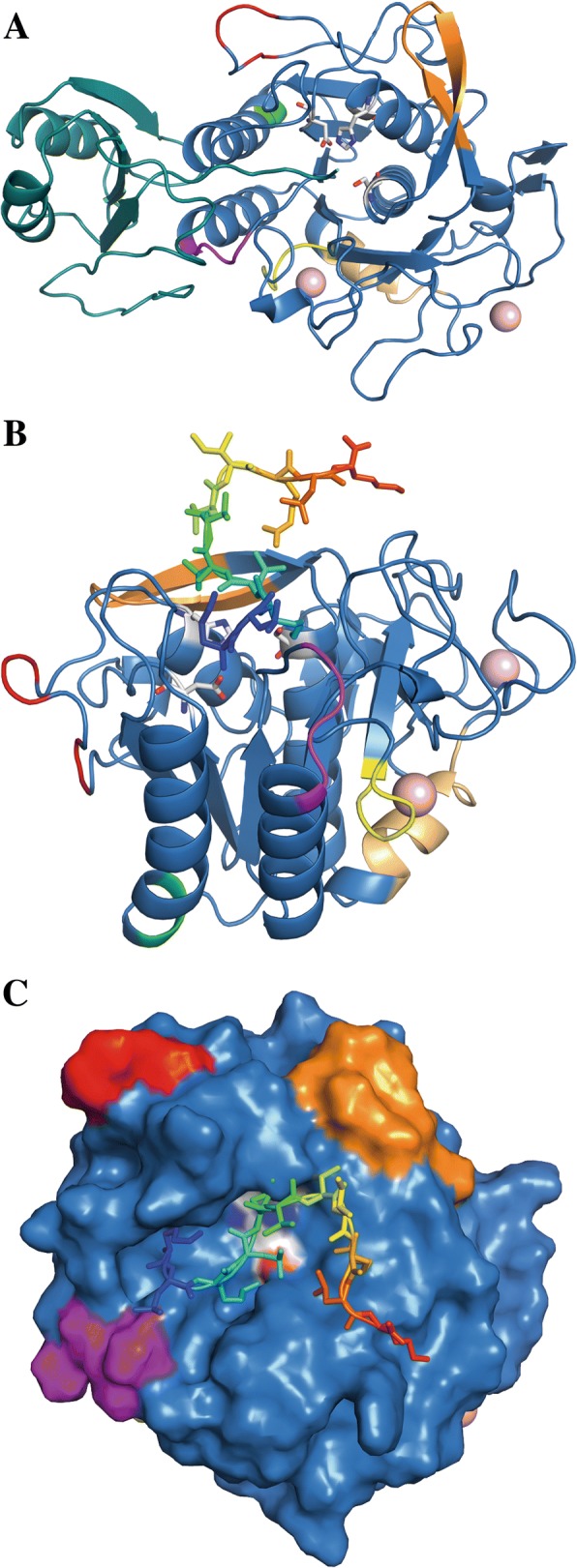
Homology model of *C. rosea* S8A serine protease BN869_T00009071 (PRS16), a propeptide (teal) and catalytic domain (blue) shown in the cartoon and highly variable regions are highlighted with colour in 6A to 6C figures. Variable region II is shown in red, region III in green, region IV in magenta, region V in yellow, region VI in orange and region VII in gold. Catalytic triad residues are shown in white stick representation, Ca^2+^ ligands (imported from PDB ID: 1IC6) shown as pink spheres and a peptide ligand (imported from PDB ID: 2HPS) is shown in rainbow coloured sticks. **a** shows a cartoon representation of the full protein, with the propeptide (teal) extending into the active site. Variable regions can be seen residing on surface accessible loops of the catalytic domain (blue). **b** shows a cartoon representation of the catalytic domain and peptide ligand, rotated 90° in regards to A and highlights the positions of regions III, V and VII which are located further away from the active site region. Region V comprises most of calcium-binding site 1 and VII is close and contributes a stabilising sulfhydryl bond to the calcium binding pocket. The active site is seen occupied by a ligand peptide molecule. **c** shows a surface representation of the catalytic domain with a ligand peptide, shown in rainbow coloured sticks, bound to the active site cleft and highlights the close positions of regions II, IV and VI to the active site cleft

Region I was located in the inhibitor I9 module (propeptide) while the other six regions (II to VII) were located in the protease module. The linker region connecting the inhibitor module and the protease module displayed high sequence variation in both clade I and clade Ib, indicative of low selective constraints (Fig. 5). All predicted residues important for catalysis and substrate binding were positioned in conserved regions. Protein modelling of *C. rosea* BN869_T00009071 (PRS16) showed that regions II to VII were predicted to be surface-exposed (Fig. 6). Regions II, IV and VI were predicted to be close to the substrate binding pocket (Fig. 6). Two calcium-binding sites were predicted, corresponding to the strong calcium-binding site 1 [83] and the weaker calcium-binding site 2 [84]. The first part of calcium-binding site 1, including the hallmark amino residues Pro and Val [85], was conserved in clade I but variable in clade Ib (region V). Calcium-binding site 2 was conserved in both clades.

DNA sequence alignments and REL analysis of clade I serine protease genes identified 5 sites that evolved under strong (Bayes factor ≥ 50) positive selection (Table 1), 278 sites that evolved under negative selection, and 866 sites that evolved neutrally. One of the positively selected sites was positioned in the signal peptide (position L_9_ with reference to *C. rosea* PRS16). Two sites were situated in region I (pos. S_62_ and A_72_) in the inhibitor I9 module (Figs. 5 and 6). The remaining two positively selected sites were closely located to variable regions III (pos. S_231_) and VI (pos. T_328_) predicted to be close to the substrate binding pocket (Figs. 5 and 6). An additional 7 sites displayed weak (Bayes factor 10–49) signatures of positive selection (Table 1). One of these sites was located in the inhibitor module close to region I (Fig. 5), while 5 sites were associated with the variable regions II, III and IV.

**Table 1:** Positively selected sites in serine protease S8A subgroup S08.054 genes

Amino acid position^a^	Posterior probability^b^	Bayes factor^b^
L_9_	0.80	107
S_62_	0.74	74
T_70_	0.38	16
A_72_	0.70	60
A_165_	0.53	29
N_172_	0.32	12
S_207_	0.41	18
S_231_	0.79	100
V_236_	0.59	36
S_245_	0.30	11
T_328_	0.78	90
V_377_	0.30	11

^a^Site position in reference to *Clonostachys rosea* BN869_T00009071 (PRS16)

^b^Determined by Random Effects Likelihood (REL) method

### Regulation of protease activity and serine protease gene expression

**Fig. 7:**
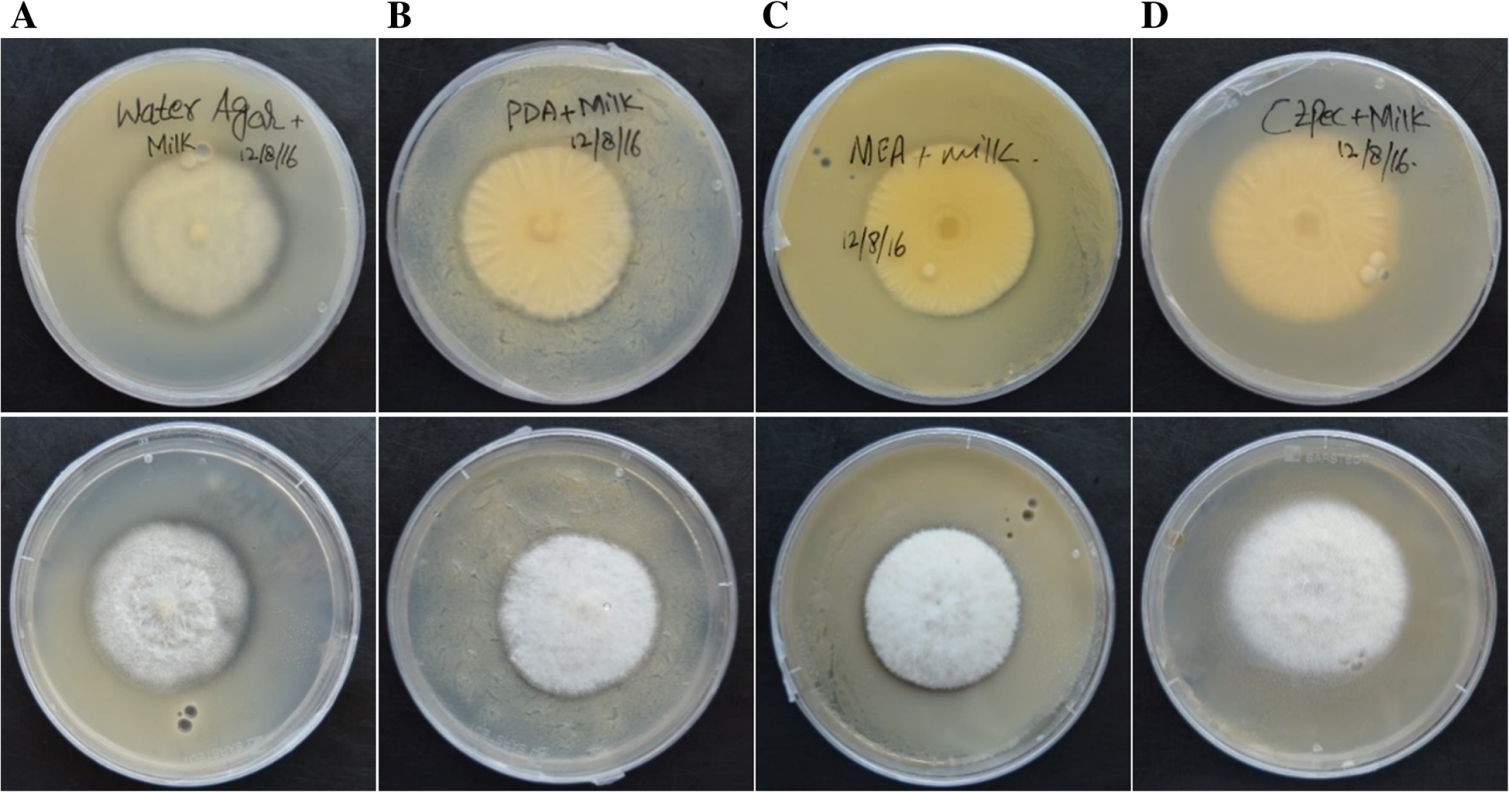
Growth of *Clonostachys rosea* on milk powder plates to assess extracellular protease activity. *Clonostachys rosea* was inoculated to plates that contained (**a**) water agar, (**b**) potato dextrose agar, (**c**) malt extract agar and (**d**) Czapek dox agar, and the clearing zones (indicative of extracellular protease activity) were assessed both from above and below the agar plates seven days post inoculation

*Clonostachys rosea* grew on all milk powder plates supplied with different growth media and formed clearing zones around and under the mycelia, indicative of extracellular protease activity, in water agar, PDA and MEA, but not when grown on CZ medium. Seven days post inoculation, the maximum width of the cleared zone was observed in plates supplied with water agar, followed by PDA and MEA (Fig. 7). The width of the cleared zone of three and 5 days post inoculation is shown in Figure S2 (Additional file 2).

Specific primers for RT-qPCR were designed (Table 2) for 18 *C. rosea* S8A serine protease paralog genes from clade I (Fig. 2). Three S8A serine protease genes, *prs2*, *prs11* and *prs14*, were repressed (*P* ≤ 0.05) during confrontations with both *B. cinerea* and *F. graminearum* (Table 3). *Prs3* and *prs13* were repressed during confrontation with *F. graminearum* but not with *B. cinerea*, while the opposite was found for *prs9* (Table 3). Only one gene *(prs6*) was induced during confrontation with *F. graminearum*, while no gene expression was detected for the *prs12* gene under the studied conditions.

**Table 2:** *Clonostachys rosea* S8A serine protease genes and oligonucleotides used for RT-qPCR gene expression analysis

Protein ID	Gene name	Forward Primer (5′ → 3′)	Reverse Primer (5′ → 3′)
BN8691_T00011169	*prs1*	CGCGGCAAGCATGGTGAAGAAG	CAACAGCCGCGCCATAGTTTGAAT
BN8691_T00008996	*prs2*	GGCCTTGCTCGCATCTCCAGT	GGCCGTTTCCGTCAGTATTGTCAC
BN8691_T00010953	*prs3*	GGCGGCCGAGCGTCTATGGTA	AAGGGCGGTGTTTCTGCTGACG
BN8691_T00008718	*prs4*	CTGCCATGGCCGGAGTCGTAG	CTTTGGGGCAGCTTCGTGTGGT
BN8691_T00008991	*prs5*	GCCCCCAGAGCACGTATTTGAGAG	CGCCGGGCTGAGTGGTGAAG
BN8691_T00008504	*prs6*	TTTCTACCCTCCCCAACAACGCTACT	ATCACTGGGGATACCGGTCAAGATA
BN8691_T00008401	*prs7*	GAACGCCCTTCGCAACCATCC	GTTCCCTCACCGGCACTACTGTCAT
BN8691_T00008478	*prs8*	GGCATTGACTCGCTCGCTCCAG	AACAGTGCCGGCAGGTATATTCTTGAC
BN8691_T00010709	*prs9*	CGGCAGCCCTCCCATCAAAC	CGGGGCAAACACGTCAACTCC
BN8691_T00002560	*prs10*	CCTCGGTGGTTTCTCTGCCTCTC	CCGCCGCCTTCCTTGTATGTG
BN8691_T00006503	*prs11*	ATGATCGCCGGAATGGAGAAGGT	CATCAATGGGGGTGCCGAAGATA
BN8691_T00012020	*prs12*	CGCCGCTGGGCTTTGTGAGT	CATTTAAGCCGCTTCGCCATTTT
BN8691_T00010529	*prs13*	CGTCGCTGCTGGCAATGGAGA	CGCCGGGGCCGAAAATGTC
BN8691_T00004335	*prs14*	CCTCGACTCCGGTGGCTCTGGTA	CCGCTGGGGACGCTGGTGAT
BN8691_T00009031	*prs15*	AAGGTCGGCGGCGGCTCTAC	CGCTGGGGACCTTCTTGATGACA
BN8691_T00009071	*prs16*	GTTTCGCCGCCAGCCTTACC	GTAGTGCCACCGGGAGACTGAGAA
BN8691_T00000462	*prs17*	ATGGGGGATTTCACGCCTTTCTTC	TTCGGGATGGGCAATAGCAATACC
BN8691_T00007432	*prs18*	CCGCCGCCATTGCTGACTC	TTGACACCGGGGGCGAAGAT

**Table 3:** Expression analysis^a^ of selected *Clonostachys rosea* S8A serine protease genes during interaction with fungal prey

	*prs1*	*prs2*	*prs3*	*prs4*	*prs5*	*prs6*	*prs7*	*prs8*	*prs9*	*prs10*	*prs11*	*prs12*	*rs13*	*prs14*	*prs15*	*prs16*	*prs17*	*prs18*
Cr-Fg	0.57 a	0.43 b	0.49 b	0.52 a	N/D	1.47 a	1.74 a	N/D	0.63 ab	N/D	0.53 b	N/D	0.48 b	0.53 b	1.54 a	0.84 a	N/D	1.48 a
Cr-BC	1.01 a	0.35 b	0.74 ab	0.77 a	N/D	1.26 ab	1.04 a	D	0.33 b	N/D	0.56 b	N/D	0.94 ab	0.24 b	0.83 b	0.71 a	1.29 a	0.98 a
Cr-Cr	1.27 a	1.08 a	1.03 a	1.13 a	N/D	1.01 b	1.24 a	N/D	1.15 a	D	1.02 a	N/D	1.10 a	1.23 a	1.12 ab	1.04 a	8.26 a	1.11 a

*N/D* no detectable expression, *D* detectable expression

^a^Gene expression of S8A serine protease genes was determined by RT-qPCR during the interaction of *C. rosea* with *B. cinerea* (Cr-Bc), *C. rosea* with *F. graminearum* (Cr-Fg) and *C. rosea* with itself (Cr-Cr, control). Relative expression is calculated as the ratio between the target gene and tubulin using the 2^–ΔΔ*C*T^ method. Different letters indicate significant differences (*P* ≤ 0.05) between treatments for each gene as determined by the *Fisher’s least significant difference (LSD) test*. *The statistical analysis was performed on a minimum of three biological replicates*

Eight S8A serine protease genes were induced (*P* ≤ 0.05) at varying levels (3 to 34-fold change) during growth on milk powder compared with the control treatment (water) (Table 4). During growth on BSA, 6 *C. rosea* genes were induced (*P* ≤ 0.05) at different levels (12 to 22-fold change) compared with the control treatment, while no genes were induced during growth on collagen (Table 4). Eight genes were induced (*P* ≤ 0.05) at varying degrees (4 to 25-fold change) during growth on BSA compared with collagen, while four genes were repressed (2 to 3-fold change) during growth on BSA compared with milk powder. Eleven genes were induced (7 to 108-fold change) during growth on milk powder compared with collagen (Table 4). Transcripts originating from *prs12* were detected in *C. rosea* during growth on milk powder.

**Table 4:** Expression analysis^a^ of selected *Clonostachys rosea* S8A serine protease genes during growth on different protein sources

	*prs1*	*prs2*	*prs3*	*prs4*	*prs5*	*prs6*	*prs7*	*prs8*	*prs9*	*prs10*	*prs11*	*prs12*	*prs13*	*prs14*	*prs15*	*prs16*	*prs17*	*prs18*
Cr-BSA	0.65 b	0.39 b	0.49 b	N/D	0.52 ab	1.33 a	N/D	N/D	0.69 a	0.53 a	0.50 ab	N/D	N/D	N/D	0.55 b	0.46 a	0.75 a	0.71 a
Cr-collagen	0.03 c	0.04 c	0.03 c	N/D	0.03 b	0.15 b	N/D	N/D	0.03 b	N/D	0.12 b	N/D	0.01 b	N/D	0.16 c	1.01 a	0.03 b	0.05 b
Cr-milk	1.00 a	1.01 a	1.01 a	D	1.02 a	1.06 a	N/D	D	1.00 a	1.03 a	1.09 a	D	1.08 a	D	1.02 a	1.02 a	1.08 a	1.01 a
Cr-water^b^	0.03 c	0.03 c	0.04 c	N/D	N/D	0.75 ab	D	N/D	0.04 b	N/D	0.37 b	N/D	N/D	N/D	0.23 bc	0.93 a	0.04 b	0.05 b

*N/D* no detectable expression, *D* detectable expression

^a^Gene expression of S8A serine protease genes was determined by RT-qPCR during growth of *C. rosea* on bovine serum albumin (Cr-BSA), collagen (Cr-collagen), milk powder (Cr-milk) or in water (Cr-water). Relative expression is calculated as the ratio between the target gene and tubulin using the 2^–ΔΔ*C*T^ method. Different letters indicate significant differences (*P* ≤ 0.05) between treatments for each gene as determined by the *Fisher’s least significant difference (LSD) test*. *The statistical analysis was performed on a minimum of three biological replicates*

^b^One biological replicate in the Cr-water treatment deviated substantially from the other four replicates and was considered as an outlier, and hence excluded from the analysis

## Discussion

Because of its mycoparasitic and nematode-parasitic lifestyle, certain strains of *C. rosea* can control both fungal [86] and nematode [87] diseases on crop plants. Although *C. rosea* proteases are implicated in parasitism of the fungal plant pathogen *H. solani* [39] as well as in nematode-parasitism [40, 82], no comprehensive analysis of the protease gene content in *C. rosea* is currently available. Therefore, we determined the protease gene content in *C. rosea* strain IK726 [38], and compared it with related mycoparasitic, nematode-parasitic, entomopathogenic and plant pathogenic species from the same order.

The highest numbers of protease genes were found in *C. rosea* and the plant pathogenic *F. solani* and *F. graminearum*, hence corresponding to the proposed common evolutionary origin of Bionectriaceae and Nectriaceae [38, 49] rather than with shared lifestyles. However, a more detailed analysis revealed that the four expanded protease families in *C. rosea*, S1A, S8A, S9X and S33, were also expanded in the mycoparasitic *T. atroviride* or *T. virens*, or in the entomopathogenic *M. robertsii*, indicating a role in ecological niche adaptation. More specifically, the expansions of the serine protease families S8A, S9X and S33 in the aggressive mycoparasites *T. atroviride* or *T. virens*, in addition to *C. rosea*, but not in the nematode-parasitizing *H. minnesotensis*, suggests that certain members of these proteases are coupled to the mycoparasitic lifestyle. In fact, the S8, S9 and S33 serine protease families are reported to be expanded in *T. atroviride* and *T. virens* compared with *T. reesei* [23, 25]. The gene losses among S8A, S9X, S12 and S33 serine proteases observed in *T. reesei* likely reflect the current life style transition, from a mycoparasitic ancestor to a wood-degrading saprotroph, in this species [23, 88], thereby providing additional support for their involvement in mycoparasitism. Several *Trichoderma* serine proteases are also reported to influence the mycoparasitic activity [27, 28, 31].

It should be noted that the opportunistic lifestyle of *C. rosea* and certain *Trichoderma* species also include nematode antagonism, for example in *T.* cf. *harzianum*, *T. atroviride* [89] and *T. longibrachiatum* [29]. Gene disruption of the *prC* S8A serine protease-encoding gene in *C. rosea* results in attenuated virulence against nematodes [40, 90], thereby establishing a link between this expanded protease family and nematode virulence in *C. rosea*. Overexpression of the S8A subtilisin-like serine protease PRB1 in *T. atroviride* resulted in a mutant that is able to penetrate egg masses and eggs of the root knot nematode *M. javanica* [88], while the serine protease SprT from *T. longibrachiatum* and the trypsin-like serine protease PRA1 from *T.* cf. *harzianum* reduced egg hatching of *M. incognita* [29, 30]. Although the nematode-parasitizing *H. minnesotensis* is reported to have fewer subtilases than the entomopatogenic *M. robertsii* [20], S8A serine proteases are reported to be expanded in the more distantly related nematode-trapping fungi from the Orbiliales order [21].

The non-random increase in gene copy number of S1A, S8A, S9X and S33 serine proteases in *C. rosea* may be due to selection for higher protease levels or to selection for functional diversification, resulting in isozyme families of members that catalyse the same biochemical reaction but differ in regulation or properties. If functional diversification is the driver for protease family expansions in *C. rosea*, we expect close paralogs to differ in structure and/or regulation [26]. As our phylogenetic and reconciliation analyses indicated that a majority of the gene gains in the *C. rosea* S8A family took place in a specific S08.054 proteinase K subgroup, we analysed sequence divergence among these paralogs in detail. Our analyses indeed identified 7 regions displaying sequence divergence between S08.054 paralogs, and the presence of sites evolving under positive selection in 5 of these regions indicates that diversifying selection is contributing to the evolutionary trajectories of serine proteases in *C. rosea*. As expected, this sequence diversification is not related to predicted residues important for catalysis and substrate binding, but is associated with surface-exposed regions. Some of these regions are also predicted to be close to the substrate binding pocket or a calcium-binding site, suggesting that the observed sequence diversification may influence functional properties of the enzymes. Amino acid residue differences in the substrate binding sites of two nematode cuticle-degrading serine proteases resulted in enzymes that differed in their hydrolytic activity towards synthetic peptide substrates [91].

The first indication of regulation of protease activity in *C. rosea* comes from the differential ability of different growth media to induce protease activity in our enzymatic assay, where water agar and PDA were the strongest inducers. The expression of *prC* in *C. rosea* is previously reported to be induced by low nitrogen levels [40, 92], which is in line with the lack of protease activity during growth on CZ medium that have a higher level of nitrogen (3 g/l) in form of sodium nitrate compared with water agar and PDA medium.

Functional diversification of S08.054 serine protease paralogs in *C. rosea* is further supported by patterns of differential gene expression between paralogs. The *C. rosea* strain used in the current work, IK726, is previously reported to upregulate several serine protease genes during parasitism of the silver scurf pathogen of potato, *H. solani* [39], including the proteinase K-like serine protease genes *prs11, prs14* and *prs16*, and the subtilase-type serine proteases BN869_T00008046 and BN869_T00008490. In contrast, the *prs11* and *prs14* genes, as well as *prs2*, *prs3*, *prs9* and *prs13*, are repressed during interaction with either *B. cinerea* or *F. graminearum* in the current work. This is most likely explained by the difference in timing of the two experimental systems; the interaction with *H. solani* reported by Lysøe et al. [39] represented an advanced stage of parasitism 7 to 28 days after co-inoculation of the two fungi, while we investigate early responses, even before physical contact, between *C. rosea* and the fungal prey. It is likely that many proteases are involved in the degradation and nutrient release from the killed fungal prey [93–95], but certain proteases may additionally have a role in sensing of the fungal prey. Several proteases and oligopeptide transporters are reported to by induced in *T. atroviride* and *T.* cf. *harzianum* before and during contact with fungal prey [96–99], leading to the hypothesis that oligopeptide fragments released from the cell wall of fungal prey species is recognized by *Trichoderma* receptors and acts as signalling molecules [98]. The *prb1* protease gene in *T. atroviride* is induced even before hyphal contact with *R. solani*, and overexpression resulted in mutants that protected cotton seedlings better than the wildtype *T. atroviride* strain [27]. In fact, the induction of the *prs6* gene during physical contact between *C. rosea* and *F. graminearum* is compatible with such a sensing function of the PRS6 protein. A low level of the *C. rosea* PrC serine protease is necessary for the subsequent induction of the *prC* gene by the presence of nematode cuticle [40], providing further support to this idea. In this context, it is interesting to note that the mycotoxin deoxynivalenone produced by *F. graminearum* is shown to suppress N-acetyl-β-D-glucosaminidase gene expression in *T. atroviride* [100], suggesting a possible involvement of the fungal prey species in actively suppressing serine protease gene expression in *C. rosea*.

Growth on different protein sources resulted in complex transcriptional patterns of S8A serine protease genes in *C. rosea*. Although there is evidence for similar regulation of closely related paralogs, for example induction of *prs17* and *prs18* during growth on BSA and milk powder, there are also several examples of close paralogs with different patterns of expression; *prs15* vs. *prs16*, *prs3* vs. *prs4* and *prs9* vs. *prs6*/*prs7*/*prs8*. These differences in regulation are not due to the presence of pseudogenes, as all investigated S8A genes are expressed in at least one condition. Collagen was included in our assay as it is a major constituent of the nematode cuticle, but unexpectedly, none of the investigated S8A serine protease genes are induced during growth on collagen. In contrast, the *prC* gene from *C. rosea* strain 611 (possibly an ortholog to *prs14*) is induced by addition of nematode cuticle material to the growth medium [40]. Nematode cuticle material also induces subtilisin-like serine proteases in the nematophagous fungus *M. haptotylum* [101]. It is possible that additional factors than collagen present in the nematode cuticle is necessary for the induction, or that intrinsic differences between the *C. rosea* strain IK726 (from Denmark) and strain 611 (from China) explains the difference in transcriptional responses.

## Conclusions

We demonstrated that S1A, S8A, S9X, and S33 serine proteases evolve non-randomly in the mycoparasitic and nematode-parasitic fungus *C. rosea*. Gene gains for S8A, S9X, and S33 serine proteases in other mycoparasitic species from the same order suggests an involvement of these proteases in biotic interactions. Regulatory and predicted structural differences between *C. rosea* S8A paralogs indicate that functional diversification is driving the observed increase in gene copy numbers. Induction of *prs6* expression in *C. rosea* during confrontation with *F. graminearum* suggests an involvement of the corresponding protease in fungal-fungal interactions, possibly releasing oligopeptide fragments as part of a sensing mechanism.

## Additional files

Additional file 1: **Table S1.** Protease genes in different fungal genomes arranged according to MEROPS peptidase database. **Table S2.** Protease inhibitor genes in different fungal genomes arranged according to MEROPS peptidase database. **Table S3.** Protein IDs used to construct S8A phylogenetic tree. **Table S4:** Presence of secretion signal peptide in serine protease subfamily S8A members. **Table S5.** Protein IDs used in reverse conservation analysis. (XLSX 46 kb)

Additional file 2: **Figure S1.** Alternative phylogenetic placements of *Clonostachys rosea* in Hypocreales. The alternative topologies were retrieved from the literature, while branch lengths were determined based on a four-gene alignment including actin, glyceraldehyde 3-phosphate dehydrogenase, DNA-directed RNA polymerase II subunit B and translation elongation factor 1 alpha, using MEGA ver. 6. The species phylogeny was calibrated to the fossil record by setting the split between *H. minnesotensis* and *H. sinensis* to 29 million years. (A) *Clonostachys rosea* (Bionectriaceae) as sister taxon with *Fusaria* (Nectriaceae) and (B) *C. rosea* (Bionectriaceae) as basal lineage in Hypocreales. The included species represents different families within the order Hypocreales: *H. minnesotensis*, *H. thompsonii* and *H. sinensis* (Ophiocordycipitaceae), *M. robertsii* (Clavicipitaceae), *T. atroviride*, *T. virens* and *T. reesei* (Hypocreaceae), *F. graminearum* and *F. solani* (Nectriaceae), *C. rosea* (Bionectriaceae) and the outgroup species *N. crassa* (Sordariales, Sordariaceae). E = entomopathogenic, M = mycoparasitic, N = nematode parasitic, P = plant pathogenic, S = saprotrophic. **Figure S2.** Growth of *Clonostachys rosea* on milk powder plates to assess extracellular protease activity. *Clonostachys rosea* was inoculated to plates that contained (A) water agar, (B) potato dextrose agar, (C) malt extract agar and (D) Czapek dox agar, and the clearing zones (indicative of extracellular protease activity) were assessed both from above and below the agar plates three and five days post inoculation. (DOCX 2101 kb)

## Abbreviations

*B. cinerea*: Botrytis cinerea
BLASTp: Basic Local Alignment Search Tool program
BSA: Bovine Serum Albumin
*C. rosea*: *Clonostachys rosea*
C-score: Confidence score
CZ: Czapek Dox agar
E-flask: Erlenmeyer flask
*F. graminearum*: *Fusarium graminearum*
*F. solani*: *Fusarium solani*
*H. minnesotensis*: *Hirsutella minnesotensis*
*H. sinensis*: *Hirsutella sinensis*
*H. solani*: *Helminthosporium solani*
*H. thompsonii*: *Hirsutella thompsonii*
*M. haptotylum*: *Monacrosporium haptotylum*
*M. incognita*: *Meloidogyne incognita*
*M. robertsii*: *Metarhizium robertsii*
MEA: Malt Extract Agar
MEGA: Molecular Evolutionary Genetics Analysis
*N. crassa*: *Neurospora crassa*
NCBI: National Center for Biotechnology Information
PDA: Potato Dextrose Agar
PDB: ID: Protein Data Bank: ID
Pro: Proline
RCA: Reverse Conservation Analysis
REL: Random Effects Likelihood
RMSD: Root Mean Square Deviation
RT-qPCR: Reverse Transcription Quantitative Polymerase Chain Reaction
SMART: Simple Modular Architecture Research Tool
*T. atroviride*: *Trichoderma atroviride*
*T.* cf. *harzianum*: *Trichoderma* confer *harzianum*
*T. longibrachiatum*: *Trichoderma longibrachiatum*
*T. virens*: *Trichoderma virens*
TM score: Template Modelling score
Val: Valine
