# Supplementary material for: Comparative evolutionary histories of fungal proteases reveal gene gains in the mycoparasitic and nematode-parasitic fungus Clonostachys rosea
Source: BMC Evol Biol. 2018 Nov 16;18:171. doi: 10.1186/s12862-018-1291-1 (PMC6240243; doi:10.1186/s12862-018-1291-1)
Supplement: Supplementary file 2 — Figure S1. Alternative phylogenetic placements of Clonostachys rosea in Hypocreales. The alternative topologies were retrieved from the literature, while branch lengths were determined based on a four-gene alignment including actin, glyceraldehyde 3-phosphate dehydrogenase, DNA-directed RNA polymerase II subunit B and translation elongation factor 1 alpha, using MEGA ver. 6. The species phylogeny was calibrated to the fossil record by setting the split between H. minnesotensis and H. sinensis to 29 million years. (A) Clonostachys rosea (Bionectriaceae) as sister taxon with Fusaria (Nectriaceae) and (B) C. rosea (Bionectriaceae) as basal lineage in Hypocreales. The included species represents different families within the order Hypocreales: H. minnesotensis, H. thompsonii and H. sinensis (Ophiocordycipitaceae), M. robertsii (Clavicipitaceae), T. atroviride, T. virens and T. reesei (Hypocreaceae), F. graminearum and F. solani (Nectriaceae), C. rosea (Bionectriaceae) and the outgroup species N. crassa (Sordariales, Sordariaceae). E = entomopathogenic, M = mycoparasitic, N = nematode parasitic, P = plant pathogenic, S = saprotrophic. Figure S2. Growth of Clonostachys rosea on milk powder plates to assess extracellular protease activity. Clonostachys rosea was inoculated to plates that contained (A) water agar, (B) potato dextrose agar, (C) malt extract agar and (D) Czapek dox agar, and the clearing zones (indicative of extracellular protease activity) were assessed both from above and below the agar plates three and five days post inoculation. (DOCX 2101 kb) [file 12862_2018_1291_MOESM2_ESM.docx]

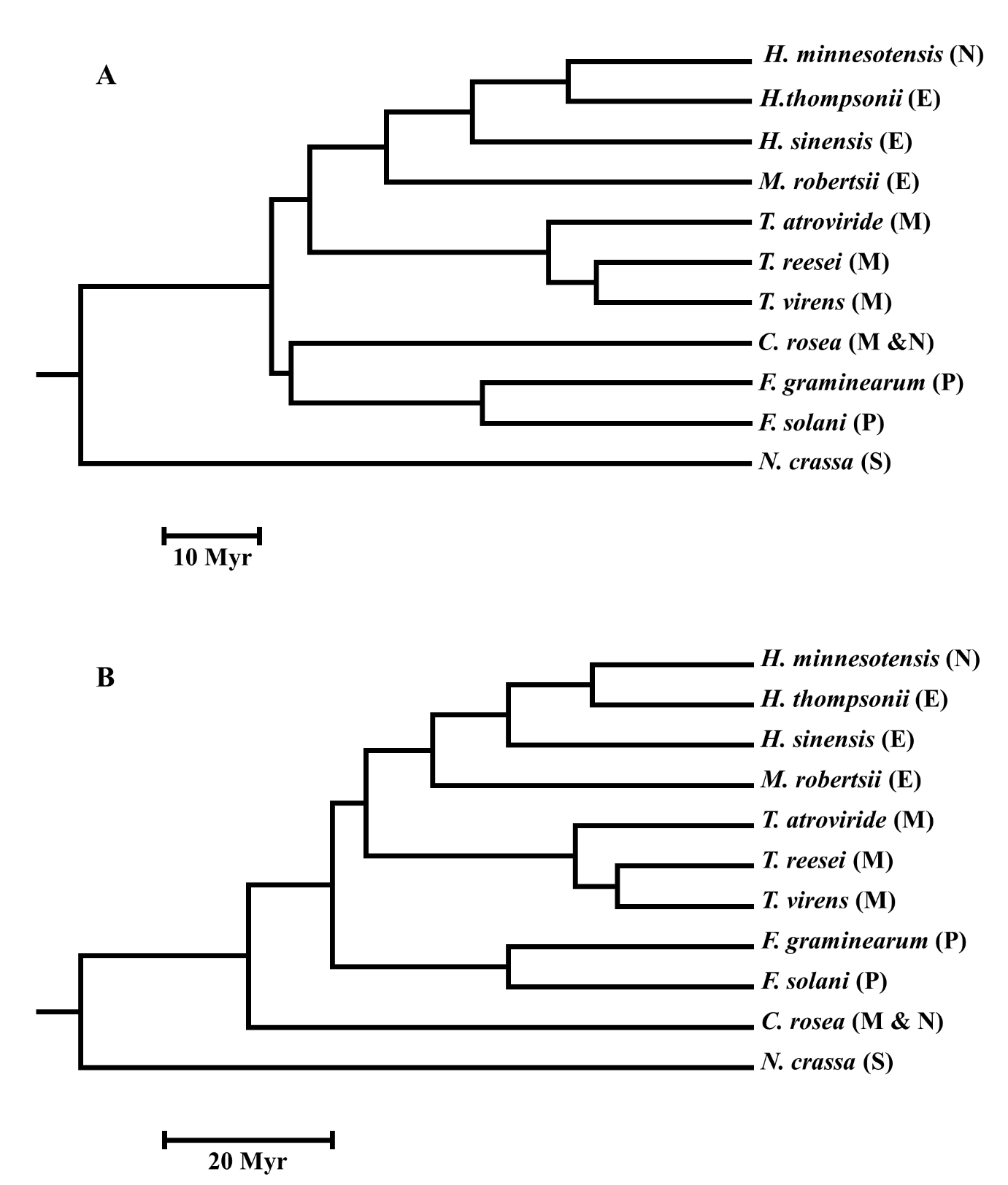


**Figure S1:** Alternative phylogenetic placements of Clonostachys rosea in Hypocreales. The alternative topologies were retrieved from the literature, while branch lengths were determined based on a four-gene alignment including actin, glyceraldehyde 3-phosphate dehydrogenase, DNA-directed RNA polymerase II subunit B and translation elongation factor 1 alpha, using MEGA ver. 6. The species phylogeny was calibrated to the fossil record by setting the split between H. minnesotensis and H. sinensis to 29 million years. (A) Clonostachys rosea (Bionectriaceae) as sister taxon with Fusaria (Nectriaceae) and (B) C. rosea (Bionectriaceae) as basal lineage in Hypocreales. The includes species represents different families within the order Hypocreales: H. minnesotensis, H. thomposonii and H. sinensis (Ophiocordycipitaceae), M. robertsii (Clavicipitaceae), T. atroviride, T. virens and T. reesei (Hypocreaceae), F. graminearum and F. solani (Nectriaceae), C. rosea (Bionectriaceae) and the outgroup species N. crassa (Sordariales, Sordariaceae). E = entomopathogenic, M = mycoparasitic, N = nematode parasitic, P = plant pathogenic, S = saprotrophic.


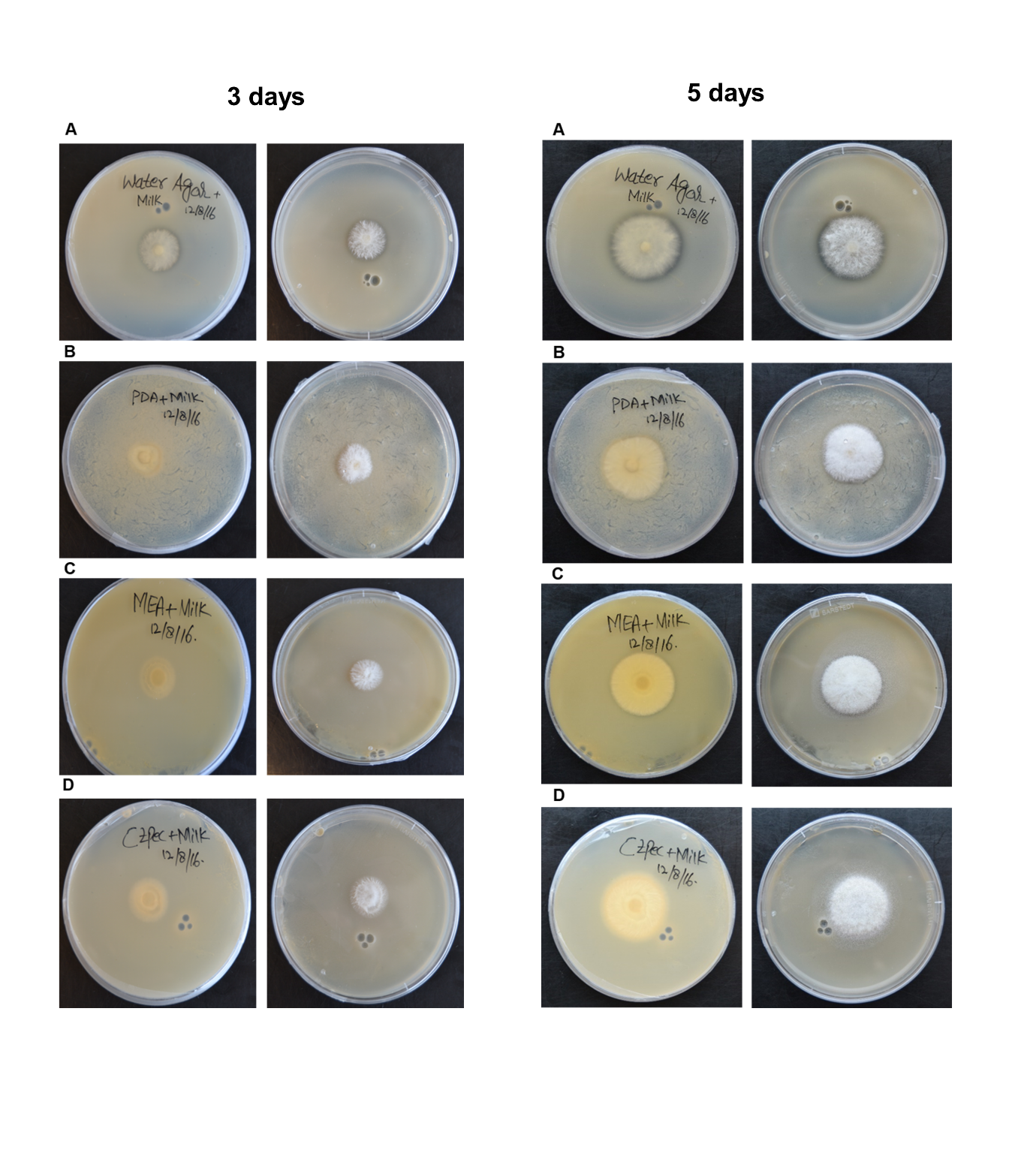


**Figure S2:** Growth of Clonostachys rosea on milk powder plates to assess extracellular protease activity. Clonostachys rosea was inoculated to plates that contained (A) water agar, (B) potato dextrose agar, (C) malt extract agar and (D) czapek dox agar, and the clearing zones (indicative of extracellular protease activity) were assessed both from above and below the agar plates three and five days post inoculation.
